# Domestic Groundwater Quality in the Northern Governorates of the West Bank, Palestine

**DOI:** 10.1155/2020/6894805

**Published:** 2020-03-28

**Authors:** Banan Hejaz, Issam A. Al-Khatib, Nidal Mahmoud

**Affiliations:** ^1^Universal Institute of Applied and Health Research, Nablus, State of Palestine; ^2^Institute of Environmental and Water Studies, Birzeit University, Birzeit, State of Palestine

## Abstract

Like several parts in the Middle East, the West Bank is in a significant water scarcity status. Palestinians use groundwater as the main water source, supplying more than 90% of the consumed water in the West Bank. The aim of this study is to enhance the knowledge on drinking water quality in the West Bank. Groundwater quality data was obtained from the Palestinian Water Authority, including the years 2015 and 2016, from the Northern six districts of the West Bank. The water quality data were analyzed and matched with the World Health Organization (WHO) guidelines and the Palestinian standards for drinking water quality. The findings of this study revealed that groundwater in the north of the West Bank comply with several drinking water requirements including total hardness, pH, and sodium and chloride content. Conversely, 18% of the samples exceed the limits for nitrate concentration. The fecal *Coliforms* and total *Coliforms* results show that 98.7% of the samples give no risk, but 1.3% of the samples give low risk, and no sample gives intermediate-to-high risks. The microbial and chemical pollution of groundwater is postulated to inadequate wastewater management, high use of fertilizers, and uncontrolled disposal of animal manure. Therefore, it is crucial to disinfect drinking water at the source of production before supply as an immediate action, followed by implementing pollution prevention measures.

## 1. Introduction

Groundwater is a priceless resource of drinking water that is used for domestic utilization, industrial activities, as well as agriculture. It is commonly of good quality as compared to other water resources due to filtration in soil [1, 2]. However, locative differences of groundwater quality are dependent on geological structure through which it flows and human activities near the groundwater basin [3]. The groundwater quality is controlled by naturalistic activities such as motion of groundwater, geology and water-rock interaction, and residence time of water in the aquifer. Also, groundwater quality is influenced by anthropogenic activities such as urbanization, industrial expansion, and agricultural activities [4–6].

While the population growth and economic development are continual, leading to water shortage worldwide, water scarcity will certainly affect urban development and food production [7]. The growing demand for using groundwater should be observed and detected to make an evaluation for the groundwater quality in addition to the quantity [8]. Definitely, pollution of groundwater is a serious environmental, social, and economic problem [9].

Water can be contaminated by microbiological, physical, and chemical pollutants, each of which is related to different causes and health-associated issues and results. Microbiological contamination of water sources is mainly caused by the improper disposal of animal and human wastes, giving rise to waterborne diseases [10]. The *Coliforms* including the fecal and total *Coliforms* are the major microbiological indicators used. The presence of these bacterial indicators in drinking water is a sign of pathogenic organisms (viruses, protozoa, parasites, and bacteria), which cause waterborne diseases [11]. Pathogenic microorganisms in water might cause waterborne illness such as typhoid, cholera, hepatitis, and respiratory system infections, as well as eye and skin diseases [12]. The chemical contamination of water is divided into organic and inorganic. Organic and inorganic chemicals originate from domestic wastewater, solid waste leachate, industrial wastewater, and agricultural runoff. Organic chemicals like the chlorinated compounds are linked to cancer, toxicity, and kidney and liver diseases. Inorganic substances, such as Boron (B), Cadmium (Cd), Molybdenum (Mo), Mercury (Hg), and Barium (Ba), may cause several diseases such as, hypertension, cancer, poisoning, and babyish cyanosis. The last is linked with the toxicity of nitrate [13]. High levels of water hardness might lead to kidney stone formation [14].

In the West Bank/Palestine, economic and population growth will result in raising the groundwater demand, as it is the major resource of water in Palestine. Deterioration of water quality in Palestine and worldwide is a key environmental challenge that requires urgent action. The aim of this study is to examine the quality of drinking water from the groundwater in the northern districts of the West Bank/Palestine. The investigated parameters include chemical, physical, and microbiological characteristics for assessing the levels of groundwater pollution.

## 2. Methodology

### 2.1. Study Area

**Figure 1:**
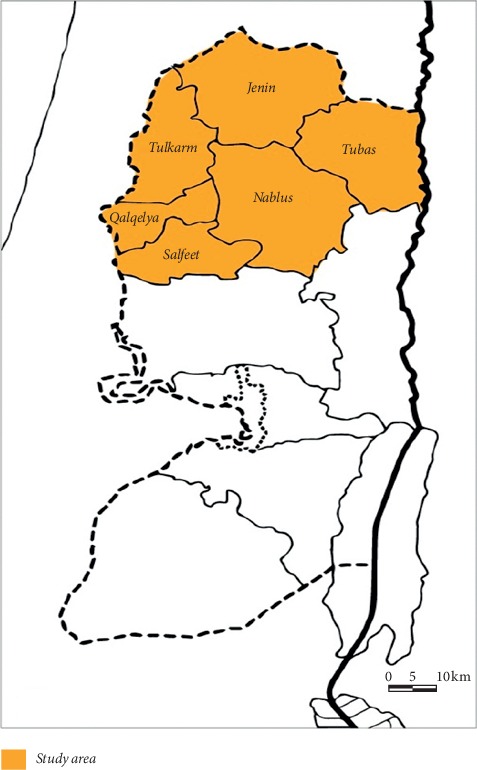
The West Bank map including the north districts [15].

**Figure 2:**
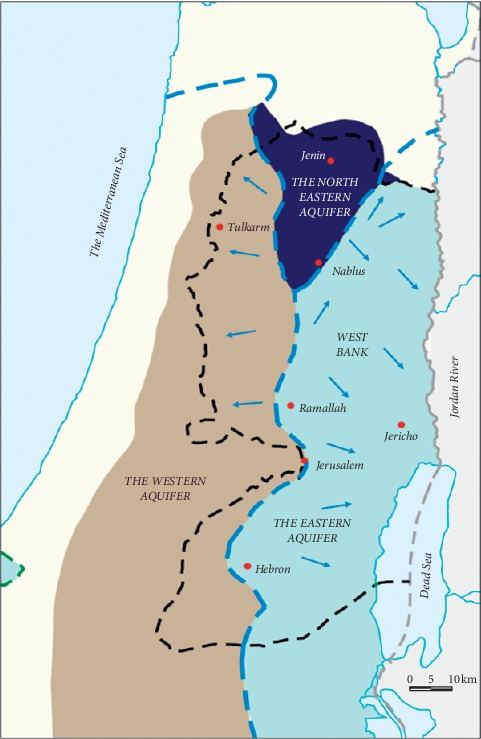
The major aquifers in the West Bank [17].

The West Bank is located in the centric mountainous land of Palestine. The region is surrounded by the Dead Sea and the Jordan River from the east and the 1948 line from the south, west, and north. It has a land area of 5,655 km^2^, with a population of 2,921,170 at the end of the year 2018. The climate of the West Bank is Mediterranean to a continental atmosphere. The study area as shown in Figure 1, includes the districts in the north of the West Bank including Nablus with a population of 388,321, Qalqilya with 112,400 persons, Jenin with 314,866 persons, Tulkarm with 186,760 persons, Tubas with 60,927 persons, and Salfit with 75,444 persons for the year 2017 [16]. Palestinians use the groundwater as the essential water source, and it supplies 90% of the water supplies and more. The major aquifer framework is separated up to three various units: the Western Aquifer Basin, the North-eastern Aquifer Basin and the Eastern Aquifer Basin for the West Bank, as shown in Figure 2.

### 2.2. Sampling and Data Collection

Groundwater quality data were collected from the official records of the Palestinian Water Authority (PWA). Water samples, with a total number of 76, were collected and tested by the PWA staff in the years 2015 and 2016 from the groundwater wells in the districts located in the north of the West Bank.

### 2.3. Water Analysis

For each water sample, the physicochemical and biological characteristics were measured using standard testing procedures [18]. Triple replicates were used in the analysis of each parameter. The investigated parameters included temperature, pH, electrical conductivity (EC), total hardness, nitrate, sodium, chloride, turbidity, and fecal and total *Coliforms*. The temperature, pH, EC, and turbidity parameters were examined in situ, using a thermometer, a portable digital pH meter, an EC meter, and a turbidity meter. For all laboratory tests, the samples were stored in 1000 mL sterile glass bottles, stored in ice box, and then sent directly to the PWA laboratories. The physicochemical parameters were tested using a DR 2400 spectrophotometer. Total and fecal *Coliforms* counts were measured by the membrane filtration technique [18].

The obtained data were recorded and categorized in tables as Microsoft Excel spread sheets for further analysis. The obtained water characteristics were then compared to the drinking water requirements set by the Palestine Standards Institution (PSI) [19] and the WHO [20]. Then, risk analysis was performed according to the obtained range of total *Coliforms* and fecal *Coliforms*.

## 3. Results and Discussion

The results of groundwater physiochemical and biological parameters are presented in Table 1 including turbidity, temperature, pH, electrical conductivity, total hardness, chloride, sodium, and nitrate, in addition to the PSI standards [19] and the WHO guidelines [20].

### 3.1. Physiochemical Parameters

#### 3.1.1. Temperature

The measured water temperature values were in the range of 18 and 27°C, with a mean value of 23°C. This is a typical temperature range within the Mediterranean region [21]. Water temperature may affect water quality through biological activities. However, the obtained temperature range is normal and thus imposes no risk [22]. Increasing the temperature of the water may affect the oxygen concentration [23].

#### 3.1.2. pH

The pH results revel that groundwater in the study area has nearly neutral to slightly alkaline characteristics, with a narrow range of 7.09–8.47. The variations in pH values could be due to geological and seasonal variations in the alkalinity of surrounding areas to springs sources [22]. These results are within the permission limits of drinking water requirements. The pH is one of the most fundamental parameters. When pH results are higher than 8, water is not proper for efficient disinfection by chlorine; however, results less than 6.5 increase attrition in the pipes. The alkaline nature of water in the West Bank reflects the dominating carbonaceous rock formations. Unlike the groundwater in the West Bank, another study was conducted in Lycoming County in Pennsylvania, USA in the year 2014, testing the groundwater quality showed wider pH range between 5.3 and 9.15, which has lower or higher levels than the standards [24]. The difference in pH values between the West Bank case and the USA case is attributed to nature differences as well as the urban and industrial activities that might lead to water acidification in the USA case.

#### 3.1.3. Turbidity

A small fraction of the samples (2.6%) was found to have turbidity values above the permission limit for drinking water. The maximum turbidity value in the tested groundwater samples is 6.4 NTU which slightly exceeds the permission limit of 5.0 NTU [20]. Turbidity is an aesthetic parameter with undefined health effect. Turbidity is imparted by solids obstructing the transmittance of light through a water sample.

#### 3.1.4. Electrical Conductivity

Electrical conductivity expresses the efficiency of water to conduct electricity, as it has a direct relation with total dissolved solids (TDS) in water [21]. The values of electrical conductivity values ranged from 401–6130 *μ*S/cm with a mean value of 820 *μ*S/cm. A small fraction of the samples (1.4%) was found to have EC values above the allowable limit for drinking water requirements. The wide variations in the EC values can be attributed to the different geological structures, agricultural activity, and soil conditions within the study area [22, 25]. Water quality is classified according to the range of EC as shown in Table 2 [26]. The majority of the tested groundwater samples is classified as good or permissible (Table 2). A small fraction of the samples (98.6%) was found to have EC values within the PSI and WHO drinking water allowable limit (2000 *μ*S/cm).

#### 3.1.5. Total Hardness

The results show that total hardness ranges between 204 and 485 mg/L as CaCO_3_ in the study area, which complies with the drinking water requirements. Water quality can be classified according to total hardness as indicated in Table 3 [21]. The groundwater classification based on the total hardness results ranges from hard to very hard. High hardness in water gives rise to extravagant consumption of soap, which is used for domestic cleaning and washing. Lowering the hardness of water is important in order to lower the quantity of soaps and detergents for domestic use [27]. Also, hard water causes scale formation in the boilers.

#### 3.1.6. Chloride

Chloride concentration in the tested groundwater is less than the MCL of 250 mg/L. Differently, Danoun [28] reported chloride concentration of 819 mg/L in the groundwater in Marj Na'ja Area in Jericho District, Palestine, which by far exceeds the drinking water requirements. The difference in chloride concentration in Jericho district as compared with the north districts of the West Bank is likely due to the difference in geographical formations. When chloride rises in water, it imparts a salty taste, and it might cause diarrhea to persons who are allergic [20].

#### 3.1.7. Sodium

Sodium concentration in the tested groundwater samples is below the MCL according to the PSI and WHO. Differently, Danoun [28] reported results in Frush Bait Dajan wells, Palestine, of high sodium concentrations ranging between 233 and 306 mg/L. Excess levels of sodium may cause obesity, absence of physical activity, and stress, and it might raise the blood pressure. The high sodium level is responsible of roughly 7 million deaths worldwide every year [29].

#### 3.1.8. Nitrate

Nitrate concentration in 18% of the tested samples exceeded the PSI and WHO drinking water requirements. This excess nitrate may cause infant methemoglobinemia [30]. Furthermore, the danger of certain cancers and childbirth disorders may be raised when nitrate is ingested under conditions that increase formation of N-nitroso compounds [31]. Danoun [28] reported high nitrate concentrations for water samples in the range of 5.2 to 45 mg/L from Marj Na'ja and Azzubied at groundwater wells and in the range of 41.8 and 114.9 mg/L from Frush Bait Dajan groundwater. The nitrate increases due to pollution with animal manure and fertilizers in the agriculture and forestry areas and also from untreated or partially treated wastewater [22]. In the West Bank, 17.1% of the households use sealed pits, while 43.3% uses porous cesspits, as well as 38.4% uses sewerage networks [16].

### 3.2. Microbiological Parameters

#### 3.2.1. Total Coliforms and Fecal Coliforms

The results of the microbiological parameters in terms of total and fecal *Coliforms* and the permissible drinking water limits are presented in Table 1. A very small fraction of the tested samples (1.3%) was found to have total *Coliforms* higher than the acceptable limit. The results of fecal *Coliforms* revealed that most of the tested groundwater samples has no fecal *Coliforms*. Only a very small fraction of the samples (1.3%) was contaminated with fecal *Coliforms*, and so not complying with the permissible limits set by the WHO [20] and the PSI [19]. The presence of fecal *Coliforms* in the tested samples indicates microbial pollution from wastewater, as well as it is possibly linked to animal flocks and their manure [32, 33].

The presence of microbial indicators in groundwater makes it unacceptable for drinking, at least without treatment.  Table 4 lists the required treatment procedures recommended by the WHO [20] for the categorized degree of contamination, according to the range of total *Coliforms.* The majority of the samples (98.7%) are not contaminated by total *Coliforms*. Only 1.3% of the samples is categorized with the first degree of contamination and so requires chlorination treatment only. Results of risk analysis of water samples are shown in Table 5. It lists the degree of risk and the percentage of tested cistern samples for fecal *Coliforms* (CFU/100 ml) according to a classified degree of risk set by the WHO [20]. Obviously, 98.7% of the tested samples impose no risks, while 1.3% imposes a moderate risk level.

## 4. Conclusions and Recommendations

The findings of this study revealed that ground water in the north of the West Bank complies with several drinking water requirements set by the PSI [17], including total hardness, pH, sodium, and chloride. Conversely, 18% of the samples exceed the limits for nitrate, which is mainly because of the inadequate wastewater management, high use of fertilizers, and uncontrolled disposal of animal manure. The excess nitrate may impose health risks due to causing infant methemoglobinemia. Nitrate should be monitored, and the sources should be carefully managed. Moreover, turbidity was higher than the limits in 1.3% of the samples tested, but it is not expected to cause any health effect. In addition to that, fecal *Coliforms* and total *Coliforms* results show that 98.7% of the samples give no risk, but 1.3% of the samples give low risk, and no samples give intermediate to high risks. The microbial pollution stresses the vital necessity to immediately disinfect drinking water before supply, followed by interventions for preventing pollution.

Here are some strategies recommended in order to limit the water crisis. First of all, it is important to assure the Palestinians water rights and then strengthen the water institutions in order to enable them to manage water sources. In order to decrease and control the pollution in the aquifers, wastewater treatment plants must be provided, fundamentally for big societies, as a first priority. Sanitation department should be improved and completed in the districts, and supervision programs must be performed to assure the suitable use of fertilizers. Furthermore, in the rural areas that are not expected to be sewered soon, emptied septage out of the cesspits must be properly handled. In addition, controlling of the industrial waste is a must.

## Figures and Tables

**Table 1:** Physiochemical and microbiological parameters of the groundwater in the Northern governorates of the West Bank, the PSI standards, and the WHO guideline.

Physiochemical parameter	Range of measured values	Avg. ± S.D	WHO guidelines [20]	PSI standards [19]	Samples over MCL^a^ of PSI (%)
Total hardness (CaCO_3_^−^ mg/L)	204–485	351 ± 60.2	NA^*∗*^	500	0%
Chloride (mg/L)	24–232	32 ± 44.7	Up to 250	Up to 250	0%
Conductivity EC (*μ*S/cm)	401–6130	820 ± 654	Up to 2000	Up to 2000	1.4%
pH	7.09–8.47	7.6 ± 0.8	6.5–8.5	6.5–8.5	0%
Sodium (Na)	21–77	32 ± 20.0	100	200	0%
Nitrate (NO_3_^−^) (mg NO_3_^−^/L)	1–82	32 ± 20.0	Up to 50	50	18%
Turbidity (NTU)	0.3–6.4	1.4 ± 1.3	Up to 5.0	Up to 5.0	2.6%
Temperature (°C)	18–27	23 ± 1.41	NA	NA	NA
Total *Coliforms* (CFU/100 mL)	0–40	0.5 ± 4.58	0	0–3	1.3
Fecal *Coliforms* (CFU/100 mL)	0–25	0.33 ± 2.87	0	0	1.3

^a^MCL: maximum concentration limit according to PSI [19]; NTU: nephelometric turbidity units; ^*∗*^NA: not available; Avg.: average; CFU: colony forming unit.

**Table 2:** Water quality classification for various ranges of EC in *μ*S/cm at 25 °C.

Range of EC (*μ*S/cm)	Water quality classification [26]	Percentage of samples (%)
<250	Excellent	0
250–750	Good	53.4
750–2,000	Permissible	45.2
2,000–3,000	Doubtful	0
>3,000	Unsuitable	1.4

**Table 3:** Water quality classification for various ranges of hardness.

Total hardness (mg/L as CaCO_3_)	Degree of hardness [21]	Percentage of samples (%)
0–75	Soft	0
75–150	Moderately hard	0
150–300	Hard	19
>300	Very hard	81

**Table 4:** Distribution of the tested groundwater samples for total *Coliforms* according to their level of contamination and recommended treatment procedure.

Recommended treatment procedure [20]	Range of total *Coliforms* (CFU/100 mL)	Degree of contamination	Percentage of samples (%)
No treatment required	0–3	0	98.7
Chlorination only	4–50	1	1.3
Flocculation, sedimentation, and then chlorination	51–50,000	2	0
Very high contamination, need special treatment	>50,000	3	0

CFU: colony forming unit.

**Table 5:** Distribution of tested groundwater samples for fecal *Coliforms* (CFU/100 mL) according to their degree of risk.

Range of fecal *Coliforms* (CFU/100 ml)	Degree of risk [20]	Number and percentage of tested samples
0	No risk	75 (98.7%)
1–10	Low risk	0 (0%)
11–100	Moderate risk	1 (1.3%)
101–1000	High risk	0 (0%)
>1000	Very high risk	0 (0%)

CFU: colony forming unit.

## Data Availability

The data used to support the findings of this study are available from the corresponding author upon request.
